# Plaque control and reduction of gingivitis: The evidence for dentifrices

**DOI:** 10.1111/prd.12257

**Published:** 2019-03-20

**Authors:** Cees Valkenburg, Fridus A. Van der Weijden, Dagmar E. Slot

**Affiliations:** ^1^ Department of Periodontology Academic Centre for Dentistry Amsterdam (ACTA) University of Amsterdam and Vrije Universiteit Amsterdam Amsterdam The Netherlands

## Abstract

This paper focuses on plaque control and the management of gingivitis in adults and summarizes the evidence of commercially available dentifrices as gathered from existing systematic reviews. Three internet sources were used to search for appropriate papers (up to and including February 2017). The search strategy was designed to include any systematic review published on dentifrices that also included an evaluation of plaque and gingivitis scores. Characteristics of the individual reviews, such as methodological aspects, quantitative data and conclusions, were extracted. The potential risk of bias was estimated and the acquired evidence was graded. Independent screening of 205 unique reviews resulted in 10 published and eligible systematic reviews. One publication evaluated the mechanical contribution of dentifrice to plaque removal. Eight papers were identified that evaluated the efficacy of a proposed single active ingredients, of which two reviewed more than one potentially active ingredient. One study compared two active ingredients. This meta‐review appraised the current state of evidence and found that toothbrushing with a standard fluoride dentifrice does not provide an added effect for the mechanical removal of dental plaque. Evidence suggests that compared with a standard dentifrice, those containing triclosan or stannous fluoride have benefits with respect to gingival health and control of dental plaque.

## 1 INTRODUCTION

Health is a valuable asset for every individual. Oral health is an integral part of general health and has a considerable effect on quality of life. A healthy dentition is one of the essential elements of oral health.^1^ In order to maintain or improve oral health, the removal of plaque and the prevention of its accumulation on the teeth and adjacent gingival tissues are needed. Mechanical and chemical methods of plaque control can prevent gingivitis.^2^ For regular personal oral hygiene, toothbrushing is the most widespread means. There is substantial evidence showing that toothbrushing and other mechanical cleansing procedures can control plaque, provided that cleaning is sufficiently thorough and performed at appropriate intervals.^3^ An essential universal recommendation by dental care professionals (DCPs) is to brush twice daily for at least 2 minutes with a fluoride dentifrice.^4^ Dentifrice is a general term used to describe preparations that are used together with a toothbrush to clean and/or polish the teeth. In daily practice, the additional application of dentifrice has been proven of value, as it is highly appreciated by its users by providing a feeling of oral freshness.^5^ Dentifrice traditionally contains abrasives in order to help remove stained pellicle and polish the teeth.^6^ In addition, it is an ideal vehicles for active ingredients employed as an oral health preventive measure. Fluoride dentifrices have been widely used for decades and remain a benchmark intervention for the prevention of dental caries.^7^, ^8^ Several formulations with specific chemical agents are marketed. Among the active agents, the following have been included in dentifrices: enzymes, amine alcohols, herbal or natural products, triclosan (Tcs), bisbiguanides (chlorhexidine [CHX]), quaternary ammonium compounds (cetylpyridinium chloride) and different metal salts (zinc salts, stannous fluoride [SnF], SnF with amine fluoride).^9^ The indications for dentifrices with active ingredients intended for patients with gingivitis are associated with long‐term use to prevent bacterial biofilm formation.

To date, two meta‐reviews have been published that evaluated the efficacy of home care regimens for mechanical plaque removal by toothbrushes and interdental cleaning devices. One meta‐review concerning the efficacy on plaque and gingivitis of mouthwash products relative to various active chemical ingredients is also available.^3^, ^10^, ^11^ To compliment this series and to complete the pursuit to summarize the current scientific evidence in support of professional recommendations, a meta‐review on dentifrices was needed. Subsequently, the purpose of this paper was to prepare a synopsis of systematic reviews concerning dentifrices with respect to managing plaque and gingivitis.

## 2 MATERIALS AND METHODS

The protocol of this meta‐review detailing the evaluation method was developed a priori following initial discussion between members of the research team. The AMSTAR (2007)^12^, ^13^ tool was used to ensure the methodological quality of the review process and improve the strength of reporting.

### 2.1 Focused patient intervention comparison outcome question

What is the evidence for an effect of dentifrices and their proposed active ingredients on plaque and gingivitis in adults, based on evidence gathered from existing systematic reviews?

### 2.2 Search strategy

For the comprehensive search strategy, three electronic databases were queried to search for appropriate papers that satisfied the study purpose. These internet sources included the National Library of Medicine, Washington, DC (MEDLINE‐PubMed), the Cochrane Library, which also includes the DARE database of systematic reviews, and the evidence database of the American Dental Association (ADA) Center for Evidence‐based Dentistry regarding home care products in the preventive dentistry category. All three databases were searched for eligible studies up to and including March 2017. The structured search strategy was designed to include any systematic review published on dentifrice products. The following strategy was used in the search of dentifrice systematic reviews: [MeSh terms/all subheadings] toothpastes OR [text words] toothpaste OR dentifrice OR toothpastes OR dentifrices. Used filter/limits: systematic review OR meta‐analysis. All reference lists of the selected studies were hand‐searched for additional published work that could possibly meet the eligibility criteria of the study. The PROSPERO database, an international database of prospectively registered systematic reviews, was checked for reviews in progress. Further unpublished work was not sought.

### 2.3 Screening and selection

Two reviewers (CV and DES) independently screened the titles and abstracts for eligible papers. If eligibility aspects were present in the title, the paper was selected for further reading. If none of the eligibility aspects was mentioned in the title, the abstract was read in detail to screen for suitability. Inclusion of titles, abstracts and ultimately full texts was based initially on full agreement between the two reviewers (CV and DES). In cases of discrepancy, the final decision was made following discussion with GAW. No attempt was made to blind the reviewers to the names of authors or the institutions and journals while making the assessment. Hand‐searching of reference lists of reviews was conducted to ensure the inclusion of additional published and potentially relevant papers. When updates of systematic reviews were published, the latest version was selected.

### 2.4 Inclusion and exclusion criteria

The inclusion criteria were:

- systematic reviews (with or without a meta‐analysis).
- no language restriction.
- reviews evaluating studies conducted in humans.
- ≥18 years old.
- in good general health.
- intervention: the use of a dentifrice in relation to plaque and gingivitis.
- data emerging from a systematic review were considered if the underlying evidence was supported by more than one original study.

The exclusion criteria were:

- patients wearing orthodontic appliances.
- patients with dental implants.

### 2.5 Data extraction and assessment of heterogeneity

The papers that fulfilled all of the selection criteria were processed for data extraction. Information extracted from the studies included publication details, focused question, search results, descriptive or meta‐analysis outcomes and conclusions. The selected systematic reviews were categorized by two authors (CV and DES) according to various active ingredients of dentifrices. Categorization was confirmed with a third author (GAW). Disagreements between the reviewers were resolved by discussion.

The heterogeneity across the included systematic reviews was detailed according to the following factors:

- study and subject characteristics.
- methodological heterogeneity (variability in review approach and risk of bias).
- analysis performed (descriptive or meta‐analysis).

### 2.6 Quality assessment

Two reviewers (CV and DES) estimated the potential risk of bias by scoring the reporting and methodological quality of the included systematic reviews according to a combination of items described by the PRISMA^14^, ^15^ guideline for reporting systematic reviews and the AMSTAR^16^ checklist for assessing the methodological quality of systematic reviews. A list of 27 items was assessed and if all individual items were given a positive rating by summing these items an overall score of 100% was obtained. Only systematic reviews including meta‐analyses could achieve a full score of 100%.^17^ The estimated risk of bias was interpreted as follows: 0%‐40% may represent a high risk of bias; 40%‐60% may represent a substantial risk of bias; 60%‐80% may represent a moderate risk of bias; 80%‐100% a low risk of bias.^11^

### 2.7 Data analysis

Data from systematic reviews were extracted when a minimum of two selected comparisons could be included for the meta‐analysis. Data of these meta‐analyses were ordered for plaque and gingival health by index. It was determined a priori to perform further analyses. This was only carried out when a minimum of two representative meta‐analyses of an active dentifrice ingredient was available and provided data as evaluated according to the same clinical index. An overall weighted mean based on the difference of means (DiffM) of the meta‐analysis, SDs and the 95% confidence interval (CI) of the weighted mean for the scores were calculated. Using the IBM SPSS Statistics for Windows, Version 22.0. (released 2013; IBM Corp., Armonk, NY, USA). Each DiffM was assigned a weight by the number of included comparisons in the meta‐analysis of the underlying systematic review.

### 2.8 Grading the body of evidence

The Grading of Recommendations Assessment, Development and Evaluation (GRADE) system,^18^ as proposed by the GRADE working group, was used to grade the evidence emerging from this meta‐review of systematic reviews.^19^, ^20^ Two reviewers (CV and DES) rated the quality of the evidence, as well as the strength of the recommendations according to the following aspects: study design, risk of bias, consistency and precision among outcomes, directness of results, detection of publication bias and magnitude of the effect.

## 3 RESULTS

### 3.1 Search and selection results

**Figure 1:**
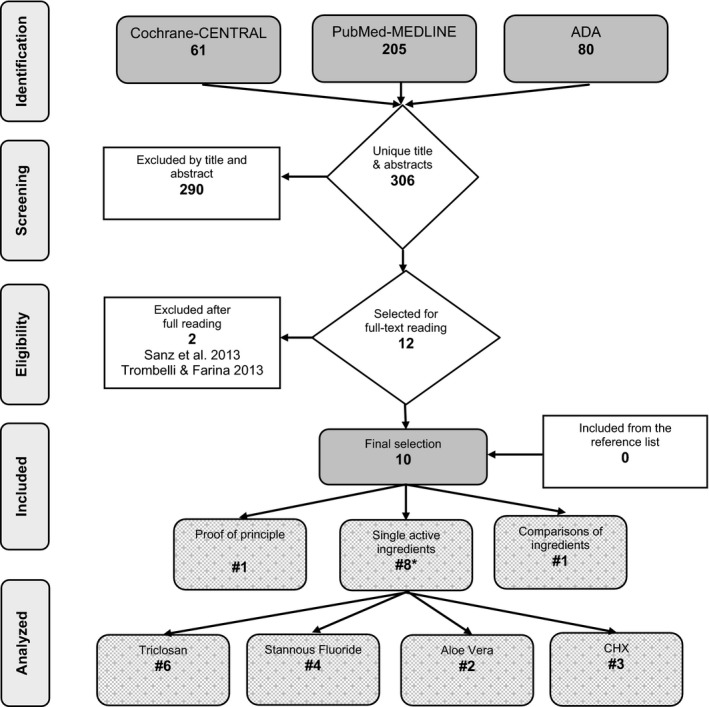
Search and selection results. *Some studies provide more than one ingredient

Figure 1 describes the search process. The searches resulted in 1806 unique titles and abstracts, out of which 12 full‐text systematic reviews were obtained and screened to confirm eligibility. Two papers were excluded because one was a narrative review^9^ and the other was a review of the literature with a systematic approach including systematic reviews of others and clinical trials.^21^ Hand‐searching of the reference lists did not reveal any additional suitable systematic reviews. Neither did the PROSPERO database.^22^ As a result, 10 systematic reviews were finally identified as being eligible for inclusion in this synopsis. One evaluated the general effect of dentifrice on plaque removal.^23^ Eight papers evaluated the efficacy of a proposed single active ingredient against a control, of which two reviewed more than one ingredient^24^, ^25^ and one systematically evaluated the comparison of two active ingredients.^10^

### 3.2 Study outcomes and assessment of heterogeneity

Heterogeneity was observed in the 10 systematic reviews with respect to the databases searched, study and subject characteristics of the original individual papers, description of inclusion and exclusion criteria, quality assessment scale used, reporting of effect scores, presence of meta‐analysis and conclusions made. For the purpose of this synopsis, a summary of the selected systematic reviews was categorized and presented by various chemical ingredients and ordered by common study characteristics.

### 3.3 Data extraction

Table 1 provides details of the extracted data for plaque index scores and gingivitis scores. DiffM, *P* values, 95% CI and tests of heterogeneity are included in these tables.

**Table 1:** Overview of data extraction of the included systematic reviews regarding plaque index scores and gingival index

Source			Index	Outcomes			Heterogeneity^a^	
Ingredient	Systematic review	No. experiments in meta‐analysis		DiffM	95% CI	*P* value	*I* ^2^, %	*P* value*
*Plaque index*								
Dentifrice	Valkenburg et al^23^	18	Q&H^28^	0.00	−0.05; 0.05	.91	0	.57
CHX	Serrano et al^25^	4	Q&H Turesky^28^	−0.687	−1.317; −0.057	.000	97.4	.000
CHX	Escribano et al^29^	3	Q&H Turesky^28^	−0.81	−1.74; 0.12	.09	98.2	?
SnF	Paraskevas and Van der Weijden^36^	4	Q&H Turesky^28^	−0.31	−0.54; −0.07	.01	91.7	<.0001
SnF	Gunsolley^24^	5	Q&H^28^	−0.168■	?	.007	?	?
SnF	Serrano et al^25^	3	Q&H Turesky^28^	−0.112	−0.185; 0.040	.002	61.4	.062
SnF	Escribano et al^29^	5	Q&H Turesky^28^	−0.28	−0.49; −0.07	.01	90.7	?
Triclosan‐COP	Serrano et al^25^	18	Q&H Turesky^28^	−0.447	−0.594; −0.300	.000	95.4	.000
Triclosan‐COP	Escribano et al^29^	16	Q&H Turesky^28^	−0.49	−0.60; −0.28	.00	94.2	?
Triclosan‐COP	Hioe and Van der Weijden^35^	9	Q&H Turesky^28^	−0.48	−0.73; −0.24	<.0001	97.2	<.00001
Triclosan‐COP	Davies et al^37^	15	Q&H^28^	−0.48	−0.64; −0.32	<.00001	95.7	<.00001
Triclosan‐COP	Riley and Lamont^79^	20	Q&H^28^	−0.47	−0.60; −0.34	<.00001	94	<.00001
Triclosan‐COP	Gunsolley^24^	18	Q&H Turesky^28^	−0.823■	?	<.00	?	?
Triclosan‐ZnCIT	Gunsolley^24^	2	Q&H Turesky^28^	?	?	.551	?	?
Triclosan‐PYRO	Gunsolley^24^	4	Q&H Turesky^28^	?	?	.040	?	?
Triclosan‐COP	Serrano et al^25^	3	S&L^32^	−0.139	−0.371; 0.094	.242	96.7	.000
Triclosan‐COP	Riley and Lamont^79^	2	L&S^31^	−0.05	−0.10; −0.01	.027	8	.30
Triclosan‐ZnCIT	Hioe and Van der Weijden^35^	6	S&L^32^	−0.07	−0.10; −0.05	<.00001	0	.53
Triclosan‐ZnCIT	Serrano et al^25^	6	S&L^32^	−0.095	−0.186; −0.005	.000	89.2	.000
Triclosan‐PYRO	Serrano et al^25^	2	S&L^32^	−0.002	−0.056; 0.060	.953	0	.739
SnF vs triclosan	Sälzer et al^10^	7	Q&H^28^	−0.29	−0.45; −0.13	<.001	90	<.001
		4	RMNPI^33^	0.09	−0.01; 0.18	.07	97	<.001
*Gingival index*								
CHX	Serrano et al^25^	4	L&S^31^	−0.289	−0.558; −0.021	.000	92.8	.000
SnF	Paraskevas and Van der Weijden^36^	6	L&S^31^	−0.15	−0.20; −0.11	<.00001	91.1	<.00001
SnF	Gunsolley^24^	6	L&S^31^	−0.441■	?	.000	?	.010
SnF	Serrano et al^25^	2	L&S^31^	−0.115	−0.161; −0.069	.000	64.8	.092
SnF AMIN	Serrano et al^25^	2	L&S^31^	−0.059	−0.074; −0.044	.000	26.5	.243
Triclosan‐COP	Serrano et al^25^	16	L&S^31^	−0.241	−0.304; −0.178	.000	91.2	.000
Triclosan‐COP	Hioe and Van der Weijden^35^	8	L&S^31^	−0.24	−0.35; −0.13	<.0001	98.3	<.00001
Triclosan‐COP	Davies et al^37^	14	L&S^31^	−0.26	−0.34; −0.18	<.00001	96.5	<.00001
Triclosan‐COP	Riley and Lamont^79^	20	L&S^31^	−0.27	−0.33; −0.21	<.00001	95	<.00001
Triclosan‐COP	Gunsolley^24^	16	L&S^31^	−0.858■	?	.000	?	<.001
Triclosan‐PYRO	Gunsolley^24^	3	L&S^31^	?	?	.647	?	?
SnF	Gunsolley^24^	2	MGI^80^	?	?	.000	?	?
SnF‐HEXA	Serrano et al^25^	2	MGI^80^	−0.382	−0.449; −0.315	.000	60.3	.112
SnF‐SHMP	Serrano et al^25^	2	BOP^81^	−4.666	−6.984; −2.347	.000	82.5	.017
Triclosan‐COP	Serrano et al^25^	2	BOP^81^	−3.153	−9.128; 2.821	.301	65.8	.087
Triclosan‐PYRO	Serrano et al^25^	2	BOP^81^	−4.344	−12.366; 3.677	.288	77.4	.036
Triclosan‐ZnCIT	Hioe and Van der Weijden^35^	4	BOP^81^	−10.81	−12.69; −8.93	<.00001	0	.48
ZnCIT	Serrano et al^25^	5	BOP^81^	−9.301	−12.875; −5.727	.000	76.8	.002
SnF vs triclosan	Sälzer et al^10^	14	L&S^31^	−0.04	−0.11; 0.04	.34	97	<.001
		7	GBI^82^	0.02	0.01; 0.03	<.001	67	.01

BOP, bleeding on probing^81^; CHX, chlorhexidine; COP, copolymer; GBI, Gingival Bleeding Index^82^; L&S, Loë‐Silness gingivitis index^31^; MGI, Modified Gingival Index^80^; RMNPI, Rustogi Modification of Navy Plaque Index^33^; S&L, Silness‐Löe plaque index^32^; SnF, stannous fluoride; Q&H, Quigley and Hein plaque index^28^; ZnCIT, zinc citrate; ?: unknown; ■: standardized mean difference; PYRO, pyrophosphates; AMIN, amin fluoride; HEXA, hexametaphosphate; SHMP, sodium hexametaphosphate.

*P value > 0.1 not significant.

Heterogeneity within the meta‐analysis can be tested by chi‐squared test and *I* ^2^ statistic. A chi‐squared test resulting in *P* < 0.1 was considered an indication of significant statistical heterogeneity. As a rough guide for assessing the possible magnitude of inconsistency across studies, an *I* ^2^ statistic of 0%‐40% can be interpreted as not important; above 40% moderate (40%‐80%) to considerable (>80%) heterogeneity may be present.^83^

### 3.4 Mechanical effect of dentifrice

Recently Valkenburg et al^23^ evaluated in a systematic review of the efficacy of brushing with or without a dentifrice for dental plaque removal. The search retrieved 10 eligible publications that included 20 comparisons. On average, 49.2% of plaque was removed when brushing was performed with a dentifrice and 50.3% of plaque was removed when toothbrushing was performed without a dentifrice. The descriptive analysis indicated that the majority of the comparisons did not show an adjuvant mechanical effect of dentifrice use. Meta‐analysis revealed that the DiffM of post‐brushing scores (DiffM), between toothbrushing with and without a dentifrice, was not significant (DiffM 0.00, 95% CI [−0.05; 0.05], *P* = .91). The meta‐analysis of incremental data (as means or percentages) supported and strengthened these findings. Based on the criteria used to rate the quality of evidence as proposed by the GRADE working group, the level of evidence was estimated to be moderate. Therefore, the authors concluded that the application of a dentifrice probably does not provide an added effect for the mechanical removal of dental plaque with a toothbrush.^23^

### 3.5 Dentifrices with proposed active ingredients

#### 3.5.1 Aloe vera

*Aloe vera* or *Aloe barbadensis miller* (family Liliaceae) is a tropical plant easily grown in hot and dry climates. Numerous cosmetic and medicinal products for different purposes contain aloe vera gel, a mucilaginous tissue found in the center of the aloe vera leaf. Aloe vera was first introduced in dentistry for healing aphthous ulcers, alveolar osteitis and lichen planus lesions. It is also suggested to have some bactericidal activity. The systematic review dedicated to aloe vera selected two randomized controlled trials. Because heterogeneity in study characteristics was evident, a meta‐analysis could not be performed. Both included studies reported that in gingivitis patients, dentifrices containing aloe vera were similar in efficacy as compared with the control dentifrices, based on the assessment of clinical, microbiological, and patient‐centered treatment outcomes. The author commented that the clinical effectiveness of aloe vera herbal dentifrices is currently not sufficiently defined and warrants further investigation.^26^

#### 3.5.2 Chlorhexidine

CHX is a cationic bisbiguanide and is active against gram‐positive and gram‐negative organisms, facultative anaerobes, aerobes and yeasts. It is often used as an active ingredient in mouthwash products designed to reduce dental plaque and oral bacteria. It has been shown to have an immediate bactericidal action and a prolonged bacteriostatic action due to adsorption onto the pellicle‐coated enamel surface. CHX is the best studied and most effective antimicrobial agent in oral care.

Slot et al^27^ evaluated the effect of CHX dentifrice/gel as compared with a regular or placebo dentifrice/gel. Screening of unique titles and abstracts resulted in 11 papers, which included 16 comparisons. Of these, nine evaluated CHX dentifrice (0.4%‐1.0%) and seven CHX gel (0.2%‐2.0%). Due to heterogeneity in the reported outcome parameters and lack of data, it was not possible to perform a meta‐analysis. A descriptive analysis revealed that with respect to plaque score reduction, the majority of the experiments using a CHX dentifrice provided a significant positive effect. Also, all studies assessing gingival bleeding as a parameter for gingivitis observed a significant reduction in favor of CHX dentifrice over placebo/control dentifrice. The combined data for CHX gel compared with a placebo did not show a trend towards a beneficial effect on plaque and bleeding scores. Two included systematic reviews evaluated the outcome with respect to end‐scores of Quigley and Hein (Q&H)^28^ plaque index in a meta‐analysis using three or four comparisons.^25^, ^29^ With a difference of one included study.^30^ The outcomes are not conclusive (Table 1). The overall weighted mean effect of CHX dentifrices as compared with a control as based on the two meta‐analyses is 0.73 (0.07) with a 95% CI (0.70; 0.79) concerning the Q&H plaque index (Table 2).^28^

**Table 2:** Summary of the calculated point estimate and measure of variability for the weighted means of the mean differences obtained from systematic reviews ordered by index and ingredient, presented compared with placebo/control as a weighed mean, SDs of the weighted mean in parentheses and corresponding 95% confidence intervals (CI)

Index	Ingredient	Number of meta‐analyses	Point estimate and measure of variability	
			Mean (SD)	95% CI
Quigley and Hein plaque index^28^	Stannous fluoride	3	0.25 (0.08)	0.20; 0.29
	Triclosan copolymer	5	0.47 (0.02)	0.47; 0.48
	Chlorhexidine	2	0.73 (0.07)	0.70; 0.79
Silness and Loe plaque index^32^	Triclosan copolymer	2	0.10 (0.05)	0.07; 0.14
	Triclosan zinc citrate	2	0.08 (0.01)	0.08; 0.09
Loe and Silness gingival index^31^	Stannous fluoride	2	0.14 (0.02)	0.13; 0.15
	Triclosan copolymer	4	0.26 (0.01)	0.25; 0.26

#### 3.5.3 Stannous fluoride

Tin fluoride, commercially commonly referred to as SnF, is a well‐known agent that has been used in dentifrice formulations since the beginning of the 1940s. Apart from a remineralization potential that has been found to reduce the incidence of dental caries, it also has antimicrobial effects. The search retrieved four systematic reviews concerning the efficacy of SnF dentifrices compared with a control paste in the prevention of plaque accumulation. On the Q&H plaque index,^28^ the meta‐analyses included between three and five comparisons; all showed a significant DiffM between −0.112 and −0.31 (Table 1). Regarding gingival health (Table 1) three meta‐analyses evaluated the effect on gingival index.^31^ All showed a significant effect as compared with a control dentifrice. The overall weighted mean effect of SnF based on three meta‐analyses was 0.25 (0.08) with a 95% CI (0.20; 0.29) for Q&H plaque index^28^ scores and from two meta‐analyses was 0.14 (0.02) with a 95% CI (0.13; 0.15) as based on the gingival index^31^ scores (Table 2).

#### 3.5.4 Triclosan

Triclosan is a nonionic chlorinated aromatic compound that has functional groups representative of both ethers and phenols. It has antibacterial and antifungal properties and is in consumer products, including soaps and detergents. Several combinations of Tcs were evaluated. Regarding Tcs/copolymer dentifrices, six meta‐analyses were found, including between nine and 18 comparisons that evaluated plaque index scores according to the Q&H plaque index.^28^ The presented DiffM varied between −0.447 and −0.49 and were significant (Table 1). The two meta‐analyses using the Silness and Löe plaque index (S&L)^32^ were inconclusive. The gingival index^31^ was evaluated in five meta‐analyses, with a DiffM that varied between −0.24 and −0.27 and was significant (Table 1). Two meta‐analyses both included six comparisons and evaluated Tcs zinc citrate dentifrice on the S&L plaque index^32^ and were significant. The calculated overall weighted mean effect on the S&L plaque index^32^ based on the two meta‐analyses was 0.08 (0.01) with a 95% CI (0.08; 0.09; Table 2). The combination of Tcs with pyrophosphate was evaluated in a single meta‐analysis on four parameters (Table 1).

Overall, the weighted mean effect of Tcs on plaque, based on the five meta‐analyses was 0.47 (0.02) with a 95% CI (0.47; 0.48) when measured according the Q&H plaque index^28^ and 0.10 (0.05) 95% CI (0.07; 0.14) based on two meta‐analyses concerning the plaque index according to Silness and Löe.^32^ Based on four meta‐analyses, 31the overall weighted mean on the gingival index^31^ was 0.26 (0.01) with a 95% CI (0.25; 0.26).

### 3.6 Comparisons of active ingredients

#### 3.6.1 Triclosan vs stannous fluoride

The systematic review by Sälzer et al^10^ included 11 publications and four unpublished reports retrieved after contacting the manufacturers of the market‐leading brands. Of the 15 studies processed for analysis, 10 were medium term and five were long term (>6 months). The meta‐analysis revealed no difference in gingival index (or its modification) between the two types of dentifrice [DiffM −0.04, 95% CI (−0.11; 0.04); *P* = .34]. The change in the average gingival bleeding score was significantly in favor of SnF [DiffM 0.02, 95% CI (0.01; 0.02); *P *< .001]. Plaque scores demonstrated a statistically significant difference in favor of Tcs, according to the Q&H plaque index^28^ (DiffM −0.29, 95% CI [−0.45; −0.13]; *P *< 0.001), but there was no difference according to the Rustogi Modified Navy Plaque (RMNPI)^33^ index [DiffM −0.09, 95% CI (−0.01; 0.18); *P *= .07].

### 3.7 Heterogeneity subanalysis

The DiffM of many reviews was associated with considerable heterogeneity, varying between 0 and 98.2%. Most reviews showed 90% heterogeneity for plaque scores of 90% and above. This was 70% and above for gingivitis scores. In cases in which heterogeneity is obvious, readers should exercise caution in their interpretation, as the DiffM may not provide an exact measure of the effect.^34^, ^35^

There was also variation in the number of underlying included studies of the selected systematic reviews concerning the Q&H^28^ plaque data. Items such as unpublished data and interpretation of data from the same population contribute to the variation in included studies. Also, the language restriction and the co‐intervention differed between selected inclusion criteria.

### 3.8 Quality assessment

Of the included systematic reviews, seven were considered to have a low estimated risk of bias (Table 3). Three reviews were estimated to have a substantial risk of bias^24^, ^35^, ^36^ and two a moderate risk of bias.^26^, ^37^ Critical items in this evaluation were the “a priori” development of a protocol and its registration, contacting authors for additional information, grading obtained evidence and addressing limitations of the systematic review.

**Table 3:** Estimated risk of bias by scoring a list of items related to the reporting and methodological quality of the included systematic reviews

Ingredient	General	Aloe vera	CHX	SnF	Triclosan			Various			Comparison
	Author										
	Valkenburg et al^23^	Dhingra^26^	Slot et al^27^	Paraskevas and Van der Weijden^36^	Davies et al^37^	Hioe and Van der Weijden^35^	Riley and Lamont^79^	Gunsolley^24^	Serrano et al^25^	Escribano et al^29^	Sälzer et al^10^
Criteria											
Current authors estimated quality score, %^a^	93	76	85	59	63	48	96	44	85	85	93
Current authors estimated risk of bias	Low	Moderate	Low	Substantial	Moderate	Substantial	Low	Substantial	Low	Low	Low

For the quality assessment score individual items with a positive rating were summed to obtain an overall percentage score.

a Items scored as reported by Sälzer et al.^84^

### 3.9 Evidence profile

Various factors were used to rate the body of evidence and strength of recommendations according to GRADE^19^, ^38^, as seen in Table 4. There is strong evidence in support of the efficacy of SnF‐ and Tcs‐containing dentifrices, which have a beneficial effect on plaque reduction as well as on gingivitis. There is weak evidence in support of the efficacy of CHX dentifrices, which have a small beneficial effect on both plaque and gingivitis scores. There is very weak evidence for a very small effect of aloe vera.

**Table 4:** Estimated evidence profile (GRADE 2011)^19^, ^38^ for the effect of various active ingredients of dentifrices on dental plaque and gingival health

GRADE	Aloe vera	Chlorhexidine	Triclosan	Stannous fluoride
Study designs	Systematic review N = 2	Systematic review N = 3	Systematic review N = 7	Systematic review N = 5
Reporting and methodological estimated potential risk of bias	Low to moderate	Low	Low to substantial	Low to substantial
Consistency	Inconsistent	Fairly consistent	Consistent	Consistent
Heterogeneity	Considerable	Considerable	Considerable	Considerable
Directness	Indirect	Direct	Direct	Direct
Precision	Imprecise	Precise	Precise	Precise
Publication bias	Possible	Possible	Possible	Possible
Magnitude of the effect	Very small	Small	Moderate	Moderate
Body of evidence	Very weak	Weak	Strong	Strong

GRADE, Grading of Recommendations Assessment, Development and Evaluation.

## 4 DISCUSSION

Chemical antiplaque agents can be employed to complement mechanical plaque removal.^11^ These agents can be incorporated into a mouth rinse, but can also be added to a fluoride dentifrice. Dentifrices have evolved and improved over the last 2000 years. The most significant improvement was the introduction of fluoride before the 1960s. This was awarded with an ADA seal of acceptance. The effect of fluoride on tooth remineralization falls beyond the scope of this review, which primarily focused on plaque and gingivitis.^39^ With respect to the efficacy of dentifrices for plaque control and in managing gingivitis, this meta‐review summarized the evidence available in the form of systematic reviews. The reason for including only systematic reviews is that this type of research generally provides more evidence than separate studies.^40^ Meta‐reviews are, in the presence of an inflationary increase in systematic reviews, the next step to give guidance to the DCP. They are also, in that sense, a step forward in the direction of a clinical guideline. Thereby, the completeness of the available evidence can be ascertained^41^ and the methodological quality of the synthesis and the clinical applicability of their findings can be assessed.^42^ Consequently, they help the clinician to find, in a timesaving manner, high‐quality information.

The evidence emerging from this meta‐review suggests that compared with a standard fluoride dentifrice, those containing Tcs or SnF have a substantial positive effect on gingival health and plaque levels. A comparison between dentifrices containing these two specific ingredients indicates that both dentifrices are clinically effective with regards to gingivitis and plaque parameters. However, with respect to plaque scores, there was inconclusive evidence in outcomes among the various indices used.

Dentifrices arguably has its most valuable role in encouraging people to clean their teeth. Most people in the developed world use dentifrice largely for cosmetic reasons. Modern developments in dentifrice formulation have led to the addition of agents to provide therapeutic, as well as cosmetic, benefits.^43^ dentifrice may, on the other hand, interfere with the perception of cleanliness due to the flavor and wetting ingredients used. However as early as 1960, Dudding et al^44^ concluded that almost 50% of people would not brush their teeth if they could not use a dentifrice. In a recent study it was also observed that brushing without the use of dentifrice was judged as unpleasant.^23^, ^45^, ^46^ For most individuals in the Western world, dentifrice provides the fresh mouthfeel and pleasant taste that make brushing an acceptable or even pleasant experience.

The ADA, as the largest national dental association, is a source of oral health‐related information for DCPs and their patients. The ADA Seal of Acceptance program began in 1931 and to this day, DCP and consumers recognize this seal as the gold standard when it comes to evaluating the safety and efficacy of dental products. All products with the ADA Seal of Acceptance have been shown to meet strict criteria for safety and effectiveness. All dentifrices with a seal contain fluoride. In addition to fluoride, dentifrices may contain active ingredients to help improve tooth sensitivity, whiten teeth or reduce gingivitis or tartar build‐up. With respect to the control of plaque and gingivitis, Tcs copolymer and SnF have been granted by an endorsement. This meta‐review showed that indeed for both ingredients a positive effect may be expected for both gingivitis and plaque scores (Table 1). In the comparison between both products, Sälzer et al^10^ concluded that given the small DiffM and inconclusive results for statistical significance for parameters of gingival health, it can be concluded that there is a minor and probably clinically insignificant difference between Tcs‐ and SnF‐containing dentifrices. Escribano et al^29^ recently performed a network meta‐analysis concerning data related to the Q&H plaque index.^28^ They presented a significant (*P *< .01) weighted mean difference of −0.34 with a 95% CI (−0.56; −0.12) between Tcs and SnF dentifrice in favor of Tcs. This is close to the DiffM as presented by Sälzer et al^10^, which was −0.29 (−0.45; −0.13) in general and −0.45 (−0.55; −0.35) for the subanalysis when only the specific brands Colgate Total^®^ (Colgate‐Palmolive Co, New York, NY, USA) and Crest Pro‐Health^®^ (Procter & Gamble Co, Cincinnati, OH, USA) were taken into account. In addition, the 95% CI show a considerable overlap. Consequently, it can be concluded that the data of Sälzer et al^10^ are supported by the latest network meta‐analysis.^29^

There are case reports on contact sensitization to Tcs‐containing dentifrice leading to blistering eruption on the buccal mucosa and lips.^47^ Tcs is an antimicrobial substance, which apart from being a dentifrice ingredient, is also added to many other household products such as soap, antiperspirant, toys and kitchen utensils (Food and Drug Administration, Scientific Committee on Consumer Safety SCCS).^48^, ^49^ Concerns regarding environmental and health effects have been extensively discussed.^48^, ^50^, ^51^ Therefore, the use of Tcs has been restricted in countries in Europe and most recently in the USA.^52^ There is, however, no clear evidence that Tcs is hazardous to humans.^48^, ^49^, ^51^ Overall resistance rates and cross‐resistance rates in the community setting are low, although in laboratory settings resistance to Tcs and cross‐resistance to antimicrobials have been demonstrated.^51^ A recent review on the risks and benefits of Tcs‐containing soap concluded that the risk of resistance outweighs the beneficial effects of Tcs‐containing soap. The environmental burden through dentifrice use, which commonly contains 0.03% Tcs, is comparatively low.^51^, ^53^ An often‐reported adverse event of dentifrices containing SnF is staining. The systematic review comparing SnF with Tcs^10^ reported that both studies evaluating staining showed a significantly higher risk for the group brushing with a SnF‐containing dentifrice as compared with Tcs. Reportedly, this risk can be reduced by adding hexametaphosphate to the dentifrice.^54^, ^55^, ^56^

Although some systematic reviews included in this meta‐review evaluated the same ingredients, they did not all synthesize the same papers. This variation can be the result of the moment in time the search was conducted, the choice of search terms, the selected databases and/or the defined inclusion criteria. Although some reviews had a comparable approach and included the same papers, the data were analyzed in a different manner. For instance, regarding CHX, Slot et al^27^ analyzed the data in a descriptive manner, whereas Serrano et al^25^ performed a meta‐analysis. The origin of this is the way in which data for the meta‐analysis were obtained. They could be extracted just as they were presented in the original research paper^27^ or additional data retrieved by contacting the authors for additional or clarification/calculation of the data or data can be imputed.^25^ However, irrespective of the way the data were analyzed, the conclusions of Slot et al^27^ and Serrano et al^25^ are in agreement and consistent, showing the same direction of the effect.

For this meta‐review, as a summary, the weighted means of the mean differences obtained from systematic reviews are calculated and presented in Table 2. To assign more weight to the studies that carry more information for this analysis, each meta‐analysis was assigned a weight by the number of included comparisons. Weighting each meta‐analysis study by precision (1/variance) was not feasible due to missing data or asymmetric confidence intervals, a possible sign of transformed values.^57^ When means are calculated, a statistical phenomenon, regression to the mean, can occur. When repeated measurements are made, this often results in values closer to the mean. In this meta‐review, when aggregating the data, regression to the mean could be expected due to an overlap of included studies in the different systematic reviews. Duplicate publication can result in an inappropriate weighting of the study results and may result in multiple publication bias.^58^ To our knowledge, there is no method of quantitatively synthesizing the findings of individual systematic reviews in meta‐reviews to address this specific problem. As a result of this multiple publication bias, the mean effect size can be increased^59^ and confidence intervals could be altered.^60^ However, visual inspection of data and Forrest plots suggests that exclusion of data is unlikely to change the overall conclusions of this meta‐review.

CHX is used in various vehicles and concentrations in commercially available products and may be purchased by consumers as mouthwash, spray or gel. It would be ideal to incorporate CHX in a dentifrice formulation.^61^ The potential of this formulation has been demonstrated in a nonbrushing study by the use of a tooth shield to protect selected teeth from toothbrushing. The use of CHX dentifrice resulted in significantly reduced plaque accumulation and gingivitis levels compared with the placebo.^62^ Yet, the inclusion of cationic antiseptics, such as CHX, in a dentifrice formulation can pose problems because CHX can be inactivated by ingredients such as flavors and anionic detergents.^63^ The effectiveness of a CHX dentifrice or gel on plaque, bleeding and gingival inflammation was, however, found to be less than a CHX mouthwash based on a recent systematic review.^64^

In terms of novel formulations, there seems to have been a shift in emphasis of the use of dentifrices in recent years.^39^ The focus on a cosmetic effect of dentifrices improved as a result of tailored abrasives to clean and whiten teeth, ingredients to facilitate removal and prevention of extrinsic stain and flavors for the purpose of breath freshening.^65^ Extrinsic tooth surface discoloration is an esthetic problem. In patients with naturally occurring extrinsic tooth surface discoloration, a whitening dentifrice can be recommended. Subsequently, using a whitening dentifrice may reduce the need for professional dental prophylaxis for esthetic reasons.^66^ An earlier systematic review assessed the evidence on the effectiveness of commercially available anticalculus dentifrices. Anticalculus dentifrices containing pyrophosphates, zinc compounds and/or copolymers were found to be effective in significantly reducing calculus scores.^67^ Several systematic reviews evaluating ingredients related to dentine hypersensitivity are also available. The evidence as gathered in a recent systematic review^68^ has shown that dentifrices with the following active agents may result in a reduction in hypersensitivity: arginine, calcium sodium phosphosilicate, SnF and strontium. Dental erosion may contribute to dentine hypersensitivity if the progressive and irreversible loss of dental hard tissue as caused by acids results in dentine exposure.^69^ Fluoride dentifrices offer a certain degree of protection. Other potentially effective formulations with active ingredients are under study.^70^ A recent systematic review^71^ graded the quality of evidence for a role of fluoride dentifrice in relation to erosion as very low. The use of low‐abrasive dentifrices, remineralizing agents and bonding agents applied to exposed dentin are frequently advocated measures in the literature but not supported by extensive research.^69^ Lately, oral malodor is a common complaint with significant sociocultural impact.^72^ Due to very limited evidence, the potential effect of a specifically formulated dentifrice alone or in combination with a mouthwash or a tongue scraper for treating oral malodor is, in general, unclear.^72^

As reported by Sälzer et al,^53^ a detergent that is added to most dentifrices can also cause side effects. Today the most widely used detergent in dentifrice is sodium lauryl sulphate (SLS). In general, people appreciate the SLS‐containing dentifrice more with respect to taste and mouthfeel.^53^ However, SLS can also be irritating to the oral mucosa in susceptible patients.^73^, ^74^ This is suggested to be related to the development of recurrent aphthous ulcers. SLS leads to an increased permeability of the mucosa, but not if Tcs or SnF is added.^75^ Also, dentifrices with a low water content have been reported to cause a mucosal response.^76^ Dentifrice without SLS seems to be beneficial in susceptible patients, in particular patients with recurrent aphthous ulcers.^73^

### 4.1 Limitations

It is common sense that a dentifrice with antiplaque agents is not designed to be employed in isolation and should be used in combination with mechanical cleaning. The latter is also effective by itself and the balance of the effect when used in adjunction is difficult to measure.^77^

- The formulation of a placebo or control dentifrice in “in vivo” antimicrobial studies is critical. Several ingredients may affect the outcome of the parameters of interest and/or the patient preferences, such as silica, SLS, flavoring agents.
- A publication bias cannot be ruled out. The included systematic reviews in general did not include unpublished studies. The results as presented in this meta‐review may therefore provide a biased estimate of the true effect (over‐estimation) because there is a tendency to publish mainly positive studies.^78^
- Other active ingredients, such as baking soda, essential oils, sanguinarine and zinc citrate, were all included in the systematic review of Serrano et al.^25^ For each, only one single study with a 6‐month duration was included. Consequently, they were excluded from this meta‐review.

### 4.2 Recommendations for further research

- Because dentifrice does not contribute with an adjuvant mechanical effect to plaque removal^23^, the evaluation of the effect on plaque regrowth seems of interest.
- Dentifrice is the most practical vehicle for delivering antiplaque and antigingivitis agents. Those with specific ingredients for gingival health are more expensive than basic regular sodium fluoride dentifrices. The long‐term use of antiplaque dentifrices would have significant cost implications. This may be prohibitive to many individuals. Therefore, a structured cost‐effective analysis seems indicated to evaluate the additional cost of these dentifrices in terms of oral health gain.

## 5 SUMMARY AND CONCLUSION

This meta‐review summarized and appraised the current state of evidence based on systematic reviews, with respect to the efficacy of various active ingredients of dentifrices regarding plaque removal and improving gingival health. Evidence suggests that compared with a standard dentifrice, those containing Tcs or SnF have substantial effects in obtaining gingival health. With respect to plaque score reduction, the results between these specific dentifrices were inconclusive.

## 6 PRACTICAL IMPLICATION

If both anticaries and antigingivitis effects are pursued based on this meta‐review, a logical choice would be to formulate a dentifrice with SnF. However, the potential staining effect prevents, at present, this to be a common recommendation.

## CONFLICT OF INTEREST

The authors declare that they have no conflict of interest. Slot and Van der Weijden have formerly received either external advisor fees, lecturer fees or research grants from companies that produce dentifrice products. Among these were Colgate, Dentaid, GABA, Lactona, Oral‐B, Philips, Procter & Gamble, Sara Lee, Sunstar, and Unilever.

## ETHICAL APPROVAL

Not required.
